# Clinical and molecular epidemiology of invasive *Staphylococcus aureus* infection in Utah children; continued dominance of MSSA over MRSA

**DOI:** 10.1371/journal.pone.0238991

**Published:** 2020-09-18

**Authors:** Hillary Crandall, Aurélie Kapusta, Jarrett Killpack, Carly Heyrend, Kody Nilsson, Mandy Dickey, Judy A. Daly, Krow Ampofo, Andrew T. Pavia, Matthew A. Mulvey, Mark Yandell, Kristina G. Hulten, Anne J. Blaschke

**Affiliations:** 1 Division of Pediatric Critical Care, Department of Pediatrics, University of Utah, Salt Lake City, Utah, United States of America; 2 Division of Pediatric Infectious Diseases, Department of Pediatrics, University of Utah, Salt Lake City, Utah, United States of America; 3 Department of Human Genetics, University of Utah, Salt Lake City, Utah, United States of America; 4 Primary Children’s Hospital, Salt Lake City, Utah, United States of America; 5 Department of Pathology, University of Utah, Salt Lake City, Utah, United States of America; 6 Department of Human Genetics, USTAR Center for Genetic Discovery, University of Utah, Salt Lake City, Utah, United States of America; 7 Division of Pediatric Infectious Diseases, Department of Pediatrics, Baylor College of Medicine, Houston, Texas, United States of America; Instituto de Technologia Quimica e Biologica, PORTUGAL

## Abstract

**Background:**

Invasive *Staphylococcus aureus* infections are a common cause of morbidity and mortality in children. In the early 2000’s the proportion of infections due the methicillin-resistant *S*. *aureus* (MRSA) increased rapidly. We described the clinical and molecular epidemiology of invasive *S*. *aureus* disease in a pediatric population.

**Methods:**

We prospectively identified children in Utah with invasive *S*. *aureus* infections. Medical records were reviewed to determine diagnosis and clinical characteristics. Isolates were genotyped using multi-locus sequence typing. The presence of genes encoding the Panton-Valentine leukocidin (PVL) was determined using polymerase chain reaction.

**Results:**

Over a 4-year period between January 2009 and December 2012, we identified 357 children, hospitalized at Primary Children’s Hospital, with invasive *S*. *aureus* infections and isolates available for the study. Methicillin-susceptible *S*. *aureus* (MSSA) caused 79% of disease, while MRSA caused only 21% of disease. Mortality associated with invasive *S*. *aureus* infection was 3.6%. The most common diagnoses were osteoarticular infections (38%) followed by central line associated blood stream infections (19%) and pneumonia (12%). We identified 41 multi-locus sequence types. The majority of isolates belonged to 6 predominant clonal complexes (CC5, CC8, CC15, CC30, CC45, CC59). PVL was present in a minority (16%) of isolates, of which most were ST8 MRSA.

**Conclusions:**

MSSA was the primary cause of invasive *S*. *aureus* infections at our institution throughout the study period. A limited number of predominant strains accounted for the majority of invasive disease. The classic virulence factor PVL was uncommon in MSSA isolates. Further study is needed to improve our understanding of *S*. *aureus* virulence and disease pathogenesis.

## Introduction

Invasive infections with *Staphylococcus aureus* are a significant cause of morbidity and mortality in children [1, 2]. Disease caused by *S*. *aureus* can range from minor skin and soft tissue infections to severe life-threatening infections with invasion into almost any anatomic site [3, 4]. Epidemiologic studies beginning in the early 2000s reported an increasing burden of invasive methicillin-resistant *S*. *aureus* (MRSA) infections in both healthy children as well as children with chronic illness [2, 5, 6]. Community acquired (CA)-MRSA has since become an established pathogen worldwide [2, 3, 6]. Several clonal lineages of CA-MRSA have been described and one virulent clone, designated USA300 by pulse-field gel electrophoresis and belonging to multilocus sequence type (ST) 8, is the dominant strain in many communities in the United States [7–9]. Some virulent strains, including USA300, carry genes for the Panton-Valentine leukocidin (PVL). Although PVL has been associated with severe disease, the role of PVL in pathogenesis remains unclear [10–15]. While there has always been considerable geographic variation in overall rates of community acquired *S*. *aureus* disease and clone types causing disease, recent epidemiologic reports in both adults and children suggest that national burden of CA-MRSA infection is declining, while rates of invasive CA-MSSA infection remain relatively unchanged [16–18]. Among children in Utah, rates of invasive *S*. *aureus* disease have remained high and relatively stable over time.

In this study we investigate the clinical and molecular epidemiology of invasive *S*. *aureus* disease at our institution, over a 4-year period, almost two decades after the emergence of CA-MRSA.

## Materials and methods

### Setting and study population

We prospectively identified all children younger than 18 years with culture-documented invasive *S*. *aureus* infection hospitalized at Primary Children’s Hospital (PCH, Salt Lake City, UT) between January 1, 2009 and December 31, 2012. PCH is a 289 bed free-standing children’s hospital that serves as both the pediatric community hospital for Salt Lake County and the only pediatric tertiary care center in the intermountain west region. PCH receives referrals from Utah, Arizona, Idaho, Wyoming, Nevada, and Montana.

Demographic and clinical information was obtained from the Intermountain Healthcare Enterprise Data Warehouse (IHC EDW). Manual review of the medical record was performed to confirm diagnosis and validate electronic data for all patients. The Institutional Review Boards of the University of Utah and Primary Children’s Hospital approved this study with a waiver of informed consent (IRB#00027819) as the data have been analyzed anonymously.

### Definitions

#### Invasive infection

Isolation of *S*. *aureus* from a normally sterile, with clinical evidence of disease at that site constituted an invasive infection. ICD-9 codes and chart review were used to determine infection site(s). Invasive infections included bacteremia, osteoarticular infections (OI), pneumonia, myositis or pyomyositis, necrotizing fasciitis, and meningitis or ventriculitis. Pneumonia cases were defined as chest imaging supportive of pneumonia and growth of *S*. *aureus* from a normally sterile (pleural fluid and/or blood) or near sterile (protected brush or bronchioalveolar lavage). Isolated skin and soft tissue infections were excluded unless associated with bacteremia.

#### Central line associated blood stream infection (CLABSI)

Children with a *S*. *aureus* blood stream infection and a central line or intravascular catheter in place >48 hours and without an infection at another site were assigned a diagnosis of CLABSI [19].

#### Toxic shock syndrome (TSS)

We defined TSS using the Centers for Disease Control and Prevention 2011 case definition: children with growth of *S*. *aureus* and fever >38.9°C, rash, post-infectious desquamation, hypotension and multisystem involvement [20].

#### Severe sepsis

Children with an invasive *S*. *aureus* infection and evidence of tissue hypoperfusion or organ dysfunction due to the infection were categorized as having severe sepsis [21].

#### Complex chronic conditions

Children with complex chronic conditions (CCC) were identified and classified using *International Classification of Diseases Ninth Revision* (ICD-9) diagnosis codes according to the schema proposed by Feudtner et al [22].

#### *S*. *aureus*-associated mortality

We defined *S*. *aureus*-associated mortality as a death within 30 days attributable to invasive *S*. *aureus* infection based on chart review. Patients who experienced an invasive *S*. *aureus* infection and survived the acute episode but died at a later date were not included in *S*. *aureus*-associated mortality.

#### Community acquired (CA), community onset healthcare associated (CO-HCA) versus hospital acquired (HA) [23]

Community acquired (CA) infections were those diagnosed within 48 hours of admission to a hospital in an otherwise healthy child. Community-onset healthcare associated (CO-HCA) infections were those diagnosed within 48 hours of admission to a hospital and occurring within one year of a previous hospitalization or in a patient with an underlying condition requiring frequent healthcare encounters. Hospital-acquired (HA) infections were those that developed greater than 48 hours after admission to the hospital.

### Identification and typing of bacterial isolates

*S*. *aureus* isolates were identified in the clinical microbiology laboratory by morphology and traditional microbiologic methods. All *S*. *aureus* isolates underwent routine antibiotic susceptibility testing by broth microdilution for oxacillin, tetracycline, trimethoprim/sulfamethoxazole, vancomycin, clindamycin, erythromycin and penicillin using Clinical and Laboratory Standards Institute methods and interpretation guidelines. Isolates were stored at -80°C until further analysis.

### Isolation of DNA and molecular typing

*S*. *aureus* isolates were inoculated into brain heart infusion broth and incubated at 37°C with 5% CO_2_ for 20 hours. Bacterial DNA was extracted using the DNeasy Blood and Tissue Kit (Qiagen, Venlo, Netherlands) for Gram-positive bacteria, per protocol, with the addition of 250 U/ml of Lysostaphin (Sigma-Aldrich, St. Louis, Missouri) during the initial lysis step. We performed next-generation sequencing of all isolates using the Ion Torrent Proton (Thermo-Fisher, Waltham, Massachusetts).

Multilocus sequence type (MLST) of isolates was determined using total genome sequencing data as previously described [24]. Sequence reads were assembled and extracted for all seven MLST loci and the arginine catabolic mobile element (ACME) using Geneious 8 (Biomatters, Auckland, New Zealand) [25]. Allelic sequences were then used to batch query the *S*. *aureus* MLST database and each isolate was assigned a sequence type and clonal complex [26]. Polymerase chain reaction (PCR) was used to detect PVL genes (*lukSF*-PV) [11]. The staphycoccal cassette chromosome *mec* (SCC*mec*) type was determined for MRSA isolates using the SCC*mec*Finder [27].

### Statistical analysis

Categorical data were compared using χ^2^ or Fisher exact test as appropriate. Continuous variables are reported as the median and interquartile range (IQR) and compared using Wilcoxon-Mann-Whitney rank-sum test. Rates and proportions were compared using χ^2^ or Fisher exact tests as appropriate. Statistical significance was set at *P* = .05, and all reported comparisons are 2-tailed. Statistical analyses were performed in STATA15 (Stata Corp, College Station, Texas).

## Results

### Patients demographics

**Fig 1:**
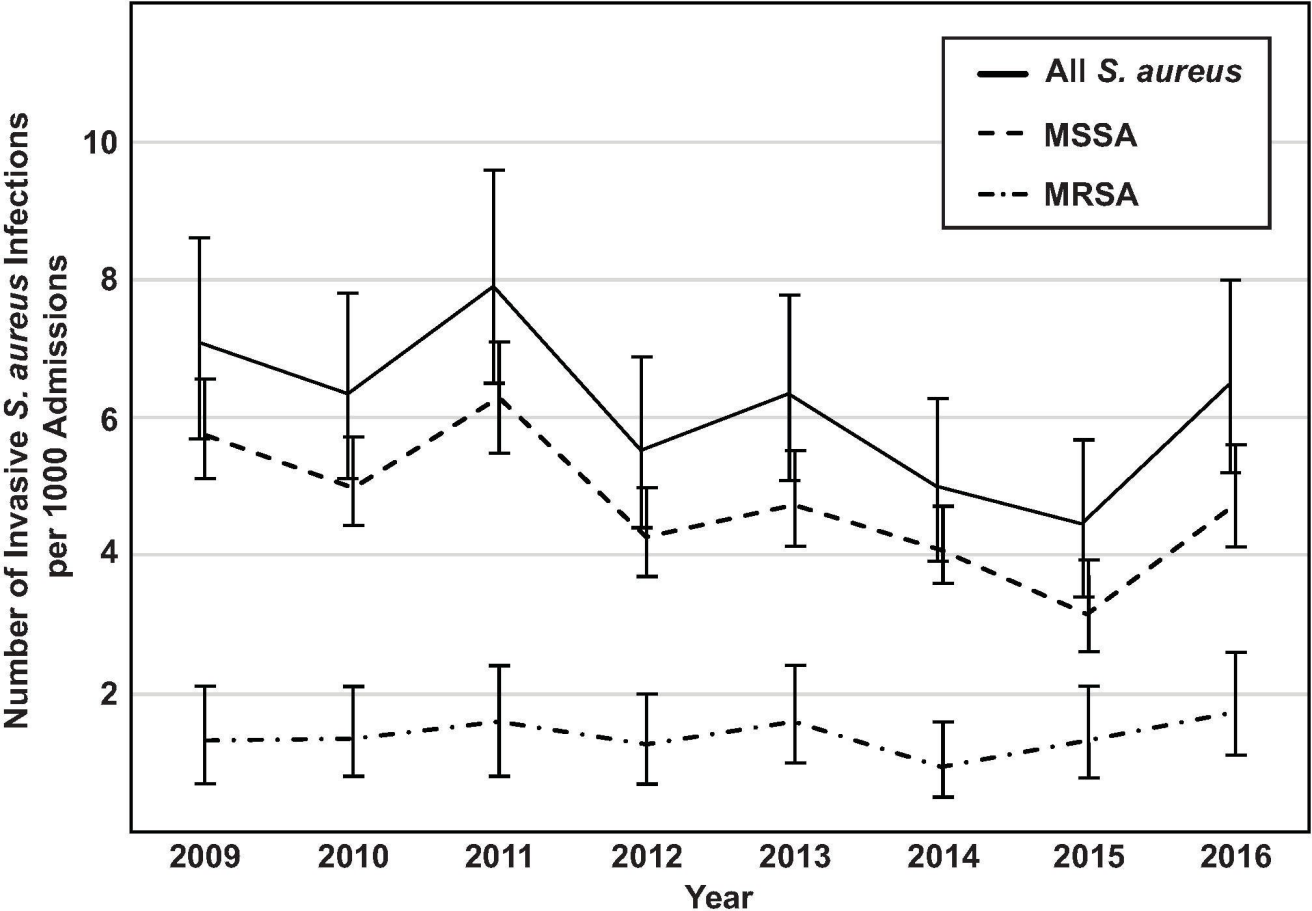
Rate of invasive *S*. *aureus* infections at Primary Children’s Hospital, 2009–2016 per 1000 inpatient admissions per year, error bars show the 95% CI.

We identified 363 children with invasive *S*. *aureus* infection treated at PCH during the 4-year study period. The rate of invasive *S*. *aureus* infection was similar across the four complete study years, with 94, 85, 103, and 81 in 2009–2012 respectively, and approximately 15 cases per 1000 inpatient admissions annually (**Fig 1**). The majority of invasive infections (283/357, 79%) were due to MSSA; the proportion was stable over the study period and has remained similar in subsequent years. Six *S*. *aureus* isolates were unavailable or failed to regrow from frozen stock, leaving 357 patient and isolate pairs included in the study.

The mean age of children with invasive *S*. *aureus* disease in was 6.7 years and did not differ between those with MSSA and those with MRSA **(Table 1**). Thirty percent of children had two or more complex chronic conditions. There was a slight male predominance overall, however, MRSA infection was associated with female gender (57% (42/73) vs. 40% (113/283); OR 2; 95%; CI 1.2–3.4; *P* = .007). There were no differences in ethnicity, or the presence of complex chronic conditions between those with MSSA and MRSA infection.

**Table 1:** Demographic characteristics of children with invasive *S*. *aureus* infection.

Characteristic	All (n = 357)	MSSA (n = 283)	MRSA (n = 74)	*P*-value
Age (years), mean ± s.d.	6.7 ± 5.7	6.8 ± 5.8	6.6 ± 5.3	0.95
Gender male, n (%)	201 (56.5)	170 (60.1)	31 (42.5)	0.007
Race/Ethnicity, n (%)				0.1
White	255 (71.4)	209 (73.8)	46 (62.2)	
Black	5 (1.4)	5 (1.8)	0	
Hispanic	45 (12.6)	32 (11.3)	13 (17.6)	
Asian	5 (1.4)	5 (1.8)	0	
American Indian Alaska Native	3 (0.84)	1 (0.35)	2 (2.7)	
Hawaiian or Pacific Islander	11 (3.1)	10 (3.5)	1 (1.4)	
Other or Unknown	33 (9.2)	21 (7.4)	12 (16.2)	
2 or more CCC, n (%)	108 (30.3)	86 (30.4)	22 (29.7)	0.53
Community Acquired	163 (45.7)	126 (44.5)	37 (50)	0.14
CO-HCA	115 (32.2)	98 (34.6)	17 (23)	
Hospital Acquired	79 (22.1)	59 (20.9)	20 (27)	

Abbreviations: MSSA, methicillin-sensitive *S*. *aureus*; MRSA, methicillin-resistant *S*. *aureus*; CCC, complex chronic conditions [22]; CoHCA, community-onset healthcare associated.

Overall, 163/357 (45.7%) of infections were community-acquired, of these 126 (77%) were MSSA and 37 (23%) were MRSA. Community-onset healthcare associated infections accounted for 32% of cases while only 22% of cases were hospital-acquired (**Table 1**). Patients with hospital-acquired infections were significantly younger than the overall study population with an average age of 2.7 ± 4.23 years (*P* = <0.001). Children with two or more complex chronic conditions accounted for 73% (79/115) of CO-HCA and 27% (29/79) of HA infections.

### Clinical manifestations and diagnose(s)

The median length of hospital stay for children with invasive *S*. *aureus* infection was seven days. (**Table 2**) More than one-third (36%) of patients required admission to an intensive care unit (ICU). Patients with MRSA has longer hospital LOS than those with MSSA (*P* = 0.008), hospital LOS was similar between the groups. Severe sepsis developed in 18% of children. Neutropenia (ANC <1500) was present, either as a pre-existing condition or during the course of infection, in 17% of children. Thirteen children (3.6%) died as a result of invasive *S*. *aureus* infection. The majority of deaths (7/13) occurred in children with one CCC, while four children who died had greater than two CCC and two children were previously healthy and had no CCC. Mortality in neonates (< 30 days of age) was 12% (4/34), accounting for 31% (4/13) of all deaths. Disease severity characteristics including length of stay, intensive care unit admission and neutropenia did not differ between those with invasive infection due to MSSA or MRSA. However, patients with MRSA had significantly greater elevation of inflammatory markers (C-reactive protein (*P* = 0.03) and erythrocyte sedimentation rate (P = 0.04)).

**Table 2:** Clinical and laboratory characteristics of children with invasive *S*. *aureus* infection.

	All (n = 357)	MSSA (n = 283)	MRSA (n = 74)	*P*-value
**Severity Characteristics**				
Hospital LOS (days), median (IQR)	7 (4–17)	6 (4–16)	10 (5–19)	0.008
ICU admission, n (%)	128 (35.9)	97 (34.3)	31 (41.9)	0.22
ICU LOS (days), median (IQR)	6.9 (2.3–19)	5.6 (2–21.3)	9.6 (3–19)	0.39
Severe Sepsis, n (%)	65 (18.2)	46 (16.3)	19 (25.7)	0.06
ANC <1500, n (%)	61 (17.1)	52 (18.4)	9 (12.2)	0.2
Mortality^c^, n (%)	13 (3.6)	9 (3.2)	5 (5.4)	0.48
**Laboratory Parameters**				
Peak WBC, 10^3^ cells/mm^3^ ± SD^a^	16.7 ± 14.4	16.5 ± 15.6	17.5 ± 8.1	0.46
Peak CRP, mg/dL ± SD^a^	16.1 ± 10.9	15.3 ± 10.6	18.9 ± 11.9	0.03
Peak ESR, mm/hour ± SD^a^	53.3 ± 35.3	50.5 ± 34.1	63 ± 38.2	0.04

Abbreviations: MSSA, methicillin-sensitive *S*. *aureus*; MRSA, methicillin-resistant *S*. *aureus*; LOS, length of stay; ICU, intensive care unit; ANC, absolute neutrophil count; WBC, white blood cell (WBC); SD, standard deviation; CRP, C-reactive protein; ESR, erythrocyte sedimentation rate.

^a^Laboratory values were not obtained on all patients, for WBC n = 350 (MSSA n = 277, MRSA n = 73), CRP n = 299 (MSSA n = 234, MRSA n = 65), ESR n = 219 (MSSA n = 171, MRSA n = 48).

Osteoarticular infections (OI) were the most common type of invasive *S*. *aureus* infection, accounting for 38% all infections. (**Table 3**) The majority (114/137) of patients with OI were previously healthy. Isolated central line associated bloodstream infection (CLABSI) accounted for 18% of infections. Pneumonia (11%) and endocarditis (3%) were less common. Infections of the central nervous system (meningitis or ventriculitis) accounted for 5.3% of invasive infections; all were associated with either a medical device (e.g. ventriculoperitoneal shunt or intrathecal pump) or trauma (e.g. basilar skull fracture). While, overall, 21% of invasive *S*. *aureus* infections were due to MRSA, MRSA was responsible for only 27 of 117 (14.6%) cases of OI. In contrast, 21 of 41 (51%) cases of staphylococcal pneumonia were due to MRSA, the majority (13/21) of which were ST8. Most children with staphylococcal pneumonia (31/41, 75%) required ICU admission. ICU admission rates were higher (*P* = 0.036) among patients with MSSA (18/20) vs. MRSA (13/21) pneumonia.

**Table 3:** Type of invasive *S*. *aureus* infection by anatomic site or syndrome.

Type of Infection, n (%)	All (n = 357)	MSSA (n = 283)	MRSA (n = 74)	*P*-value
Osteoarticular infection	137 (38.4)	117 (41.3)	20 (27)	0.024
Osteoarticular infection with bacteremia	85 (23.8)	71 (25.1)	14 (18.9)	0.27
CLABSI	66 (18.5)	56 (19.8)	10 (13.5)	0.22
Pneumonia	41 (11.5)	20 (7.1)	21 (28.4)	<0.001
Pneumonia with bacteremia	21 (12.3)	14 (12.2)	5 (12.8)	0.92
Meningitis or ventriculitis	19 (5.3)	16 (5.7)	3 (4.1)	
SSTI with bacteremia	13 (3.6)	8 (2.8)	5 (6.8)	
Endocarditis	10 (2.8)	7 (2.5)	3 (4.1)	
Mediastinitis	9 (2.5)	7 (2.5)	2 (2.7)	
Toxic shock syndrome	6 (1.7)	6 (2.1)	0	
Thrombophlebitis	5 (1.4)	3 (1.1)	2 (2.7)	
Necrotizing fasciitis	2 (0.7)	2 (0.7)	0	
Bacteremia, all sources	222 (62.2)	182 (64.3)	40 (54.1)	0.11

Abbreviations: MSSA, methicillin-sensitive *S*. *aureus*; MRSA, methicillin-resistant *S*. *aureus*; central line-associated blood stream infection, CLABSI; skin and soft tissue infection, SSTI.

### Molecular characteristics

**Fig 2:**
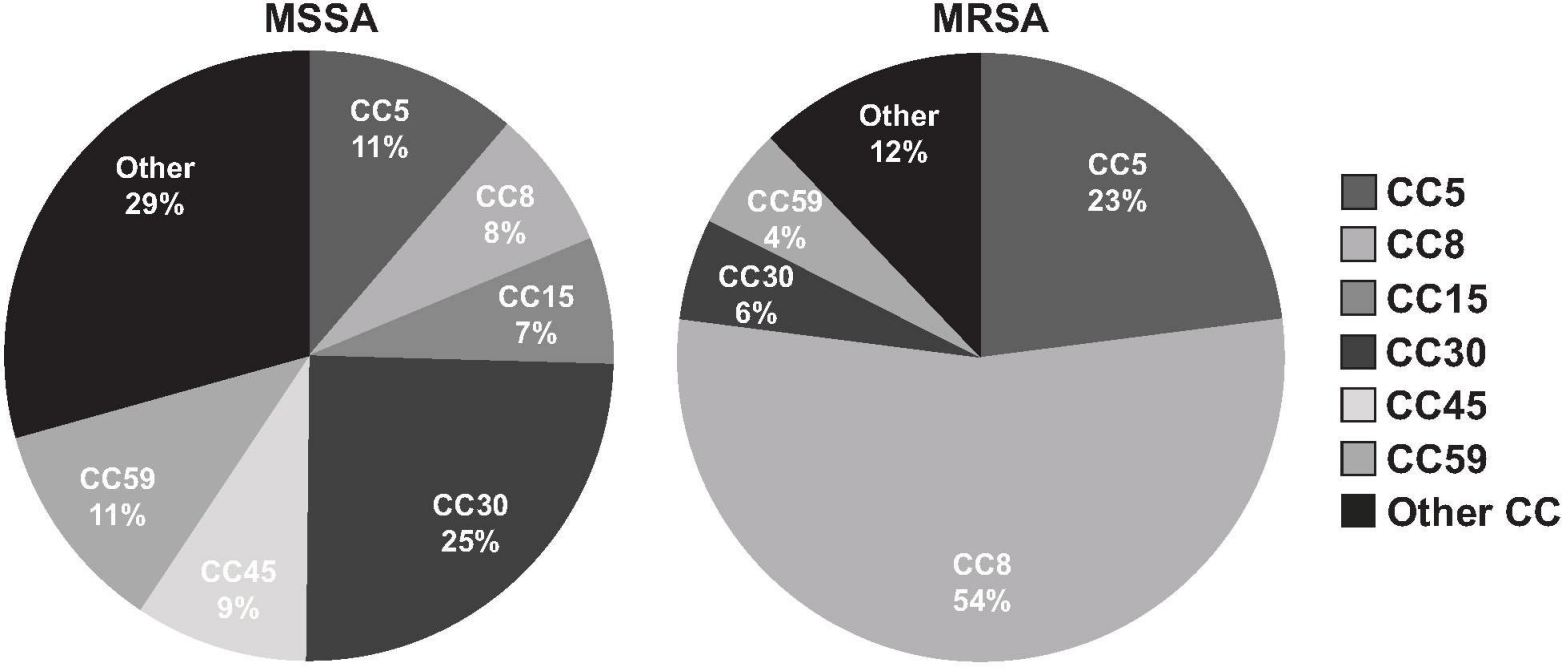
Proportional distribution of the most common *S*. *aureus* clonal complex types for MSSA and MRSA.

We identified 41 *S*. *aureus* MLST clones amongst our 357 isolates. Six distinct clonal complexes (CC), including CC5, CC8, CC15, CC30, CC45 and CC59 accounted for 68.6% of isolates causing invasive *S*. *aureus* disease (**Fig 2**). The majority of MRSA infections (40/74; 54.1%) were caused by isolates belonging to CC8, all of which were categorized as ST8. An additional large subset of the MRSA isolates (17/74; 23%) belonged to CC5. MSSA infections were predominantly caused by CC5, CC15, CC30, CC45 and CC59 isolates. ST8 MRSA isolates were more commonly associated with pneumonia (21.3% vs. 9.5%, OR = 2.6, 95% CI 1.26–5.3, *P* = .008) (**Table 4**). CC5 was significantly less commonly associated with OI than other clonal complexes (22.4% vs. 40.9%; OR = 0.42, 95% CI 0.21–0.84, *P* = .01). There were no other statistically significant associations between sequence types and specific disease manifestation(s).

**Table 4:** Clinical Presentations, outcomes and molecular characteristics of invasive *S*. *aureus* infections.

	All (n = 357)	CC5 (n = 49)	CC8 (n = 61)	CC15 (n = 19)	CC30 (n = 74)	CC45 (n = 27)	CC59 (n = 35)	Other (n = 92)
**Type of Infection, n (% of CC)**								
Osteoarticular infection	137 (37.9)	11 (22.4)	21 (34.3)	4 (21.1)	32 (43.2)	14 (51.9)	17 (48.6)	38 (41.3)
CLABSI	66 (18.5)	12 (24.5)	5 (8.2)	4 (21.1)	18 (24.3)	4 (14.8)	4 (11.4)	19 (20.1)
Pneumonia	41 (11.5)	5 (10.2)	13 (21.3)	2 (10.5)	8 (10.8)	1 (3.7)	1 (2.9)	11 (12)
Endocarditis	10 (2.8)	1 (2.0)	3 (4.9)	1 (5.3)	2 (2.7)	0	2 (5.7)	1 (1.1)
Bacteremia, all sources	222 (62.2)	29 (59.2)	38 (62.3)	10 (52.6)	49 (66.2)	13 (48.2)	24 (68.6)	59 (64.1)
**Clinical Outcomes, n (% of CC)**								
Severe sepsis	65 (18.2)	10 (20.4)	15 (24.6)	4 (21.1)	13 (17.6)	1 (3.7)	6 (17.1)	16 (17.4)
ICU admission	128 (35.9)	22 (44.9)	23 (36.1)	9 (47.4)	28 (37.8)	6 (22.2)	13 (37.1)	28 (30.4)
Mortality	13 (3.6)	3 (6.1)	1 (1.6)	1 (5.3)	3 (4.1)	1 (3.7)	1 (2.9)	3 (3.3)
**Genetic Factors, n (% of CC)**								
PVL+	56 (15.8)	0	41 (67.2)	0	1 (1.3)	0	0	14 (15.4)
MRSA	74 (20.7)	17 (34.7)	40 (65.6)	0	4 (5.4)	1 (3.7)	3 (8.6)	9 (9.8)
ACME	36 (10.2)	0	33 (55)	0	1 (1.4)	0	0	2 (2.2)

Abbreviations: CC, clonal complex; central line-associated blood stream infection, CLABSI; skin and soft tissue infection, SSTI; ICU, intensive care unit; PVL, Panton-Valentine leukocidin; MRSA, methicillin-resistant *S*. *aureus*; ACME, arginine catabolic mobile element.

The putative virulence factor PVL was only present in 16% of our isolates (56/357) (**Table 4**). Only 6% (17/283) of MSSA isolates harbored PVL, compared to 53% of MRSA isolates (39/74, *P* < .001). PVL was present in 67.2% (41/61) of ST8 isolates and 85% (34/40) of ST8 MRSA isolates. The presence of PVL was not associated with increased risk for ICU admission (19/128 vs. 37/228; OR 0.9, 95% CI 0.5–1.63, *P* = 0.73) or the development of severe sepsis (14/65 vs. 42/291, RR 1.63, 95% CI 0.84–3.2, *P* = 0.15). ACME was present in 10.2% of all isolates, the majority of which (33/36) were ST8. The presence of ACME was not associated with increased risk for ICU admission (12/127 vs. 24/227, OR 0.88, 95% CI 0.43–1.81, *P* = 0.74) or the development of severe sepsis (14/65 vs. 42/291, OR 1.89, 95% CI 0.87–4.1, *P* = 0.11). PVL and ACME were both significantly more likely to be present in ST8 isolates (P<0.001) and were rare in other STs (**Table 4**).

Antibiotic resistance to commonly used antistaphylococcal antibiotics was unusual among MRSA isolates and did not appear to change over the study period. MRSA isolates most commonly harbored SCC*mec* types IV (2B) and II (2A) (**Table 5**). Clindamycin resistance occurred in 14.9% of MRSA isolates and was associated with CC5 isolates (*P* = 0.001) but not healthcare associated infections, while resistance to trimethoprim-sulfamethoxazole was present in only 5.4% of MRSA isolates (**Table 5**).

**Table 5:** Antibiotic resistance patterns in invasive methicillin resistant *S*. *aureus* infections.

	All (n = 74)	CC5 (n = 17)	CC8 (n = 40)	CC30 (n = 4)	CC45 (n = 1)	CC59 (n = 3)	Other (n = 9)
**SCC*mec*, n (% of CC)**							
Type II (2A)	13 (17.6)	10 (58.8)	0	0	1 (100)	0	2 (22.2)
Type IV (2B)	57 (77)	7 (41.2)	39 (97.5)	2 (50)	0	3 (100)	6 (66.6)
Type V (5C2&5)	1 (1.3)	0	1 (2.5)	0	0	0	0
Unknown	3 (4.0)	0	0	2 (50)	0	0	1 (1.1)
**Other Antibiotics, n (% of CC)**							
Clindamycin	11 (14.9)	6 (35.3)	1 (2.5)	1 (25)	1 (100)	0	2 (2.2)
Trimethoprim-Sulfamethoxazole	4 (5.4)	0	1 (2.5)	0	0	1 (33.3)	2 (2.2)

Abbreviations: CC, clonal complex; SCC, staphylococcal cassette chromosome.

## Discussion

In contrast to reports from the earlier decade, we observed stable rates of invasive disease due to both MSSA and MRSA at our institution; only 21% of invasive disease was caused by MRSA [28–32]. Invasive disease due to *S*. *aureus* was severe; median length of stay was seven days, 35% of patients required ICU admission, and 4% of children died. Surprisingly, the severity of disease cause by MSSA was generally comparable to disease caused by MRSA. The majority of MRSA isolates were ST8 (USA300) and carried genes for PVL and ACME, two previously identified putative virulence factors [25, 33, 34]. In contrast, our MSSA isolates were overwhelmingly negative for PVL and ACME, and were associated with a limited number of dominant clonal types, suggesting that specific genetic factors present in these clones contribute to virulence and disease pathogenesis.

Studies in the early 2000s described the emergence of CA-MRSA as a dominant cause of invasive staphylococcal disease in certain geographic areas [2, 35]. In many communities in the US an increase in severe invasive disease was associated with specific lineages of CA-MRSA, such as ST8 (USA300) strains [8, 31, 36]. Recent reports describe significant variation in rates of MRSA disease across US medical centers, and in some areas CA-MRSA disease may be declining [9, 17, 37]. In addition, epidemiologic studies from some of these locations have reported an increasing incidence of severe invasive CA-MSSA disease [38]. For example Hulten et al., recently reported that at Texas Children’s Hospital, where USA300 CA-MRSA previously dominated, the rate of USA300 CA-MRSA-associated disease has declined accompanied by an modest increase in non-USA300 CA-MSSA disease [16]. In our patient population we observed relatively modest and stable rates of severe invasive disease caused by ST8 strains and a sustained and high rate of severe invasive MSSA infections. The severity of invasive MSSA infections in our population were generally similar to those associated with ST8 MRSA.

To understand the clonal origins of our invasive *S*. *aureus* disease we characterized isolates by MLST. Similar to reports from other locations, the majority of CA-MRSA disease in our population was caused by ST8 (USA300) isolates [8, 35, 39]. These ST8 strains had similar genetic profiles to previous reports of USA300 isolates, harboring PVL, ACME and SCC*mec* type IV [25, 40]. We identified ST8 isolates more frequently in patients with pneumonia. This is consistent with several studies that have identified ST8 (USA300) as a frequent cause of severe pneumonia indicating there may be specific genetic factors present in ST8 isolates that contribute to both virulence and tropism for the respiratory tract [29, 33]. Interestingly, although ST8 strains account for the majority of MRSA infections, they did not emerge as the predominant strain type causing invasive infection at any point in the study period. We hypothesize that this epidemiologic pattern may be due to a predominance of highly pathogenic MSSA isolates. The re-emergence of MSSA across many regions of the US suggests the need to identify factors contributing to re-emergence and virulence of MSSA not associated with methicillin-resistance or the ST8 genotype.

In our population, MSSA sorted into six predominant clonal types dominated by CC30. Several of the dominant clonal types in our study (CC30, CC45, and CC59) have been previously described as both MSSA and MRSA clones [41, 42]. In our study, CC30, CC45 and CC59 isolates were almost exclusively MSSA and were predominantly associated with OI. Although CC30 and CC45 have been reported in association with OI, an association between CC59 with OI has not previously been reported [43, 44]. Select CC30 lineages have been associated with increased rates of invasive disease, OI, and endocarditis [45]. For example, Nienaber *et al*. found that MSSA isolates causing infective endocarditis were significantly more likely to be CC30 and possess a unique set of potential virulence genes. Luedicke *et al*. found an increased proportion of CC45 isolates among patients with OI [43, 46]. Cassat *et al*. associated the presence the *cna* gene with hypervirulent OI strains in their population [47]. In contrast, Perez-Montarelo *et al*. noted increased frequency of *msrA* and *hla* in OI strains as well as *sed*, *splE* and *fib* in endocarditis strains [48]. Our study, in conjunction with these and other previous reports, suggests that there are specific genetic factors that may impact both the likelihood of hematogenous spread as well as tropism of certain *S*. *aureus* isolates for connective tissue, bone and joints.

Multiple factors may contribute to the molecular basis of *S*. *aureus* pathogenesis and virulence. Among these, the bicomponent leukotoxin PVL is perhaps the most intensely investigated. PVL has been associated with both ST8 (USA300) isolates and severe invasive infections [11, 49]. Consistent with previous reports, PVL was present in the majority of ST8 isolates in our cohort and was rare in other isolates [8, 10]. The majority of invasive infections in our population were caused by PVL-negative isolates, consistent with complex and multifactorial determinants of virulence. Other investigators have described a number of possible non-PVL *S*. *aureus* virulence factors [50]. In future investigations, we will use the full sequences of this large collection of invasive isolates from a well characterized cohort to investigate the multi-factorial virulence of *S*. *aureus*.

This study has several limitations. Our study is limited to a single center, and the distribution of *S*. *aureus* clones and their genetic composition may be different in other regions. Our study is limited to children; epidemiology may differ in adults due to differences in colonization patterns and immunity. A strength of our study is its size and completeness, as our hospital cares for the vast majority of invasive *S*. *aureus* disease in children in our region.

Our study along with other recent studies highlight the complexity of changing patterns of S. aureus epidemiology and the challenges of dissecting determinants of virulence. Continued study of the genetic commonalities and differences of these isolates as well as associations with disease phenotypes will lead to a better understanding of *S*. *aureus* virulence.

## Supporting information

S1 File (CSV)

S2 File (XLSX)
